# Myofibril and Mitochondrial Area Changes in Type I and II Fibers Following 10 Weeks of Resistance Training in Previously Untrained Men

**DOI:** 10.3389/fphys.2021.728683

**Published:** 2021-09-24

**Authors:** Bradley A. Ruple, Joshua S. Godwin, Paulo H. C. Mesquita, Shelby C. Osburn, Casey L. Sexton, Morgan A. Smith, Jeremy C. Ogletree, Michael D. Goodlett, Joseph L. Edison, Arny A. Ferrando, Andrew D. Fruge, Andreas N. Kavazis, Kaelin C. Young, Michael D. Roberts

**Affiliations:** ^1^School of Kinesiology, Auburn University, Auburn, AL, United States; ^2^Athletics Department, Auburn University, Auburn, AL, United States; ^3^Department of Geriatrics, Edward Via College of Osteopathic Medicine, Auburn, AL, United States; ^4^Department of Geriatrics, Donald W. Reynolds Institute on Aging, University of Arkansas for Medical Sciences, Little Rock, AK, United States; ^5^Department of Nutrition, Dietetics and Hospitality Management, Auburn University, Auburn, AL, United States

**Keywords:** myofibrils, mitochondria, resistance training, histology, peripheral quantitative computed tomography

## Abstract

Resistance training increases muscle fiber hypertrophy, but the morphological adaptations that occur within muscle fibers remain largely unresolved. Fifteen males with minimal training experience (24±4years, 23.9±3.1kg/m^2^ body mass index) performed 10weeks of conventional, full-body resistance training (2× weekly). Body composition, the radiological density of the vastus lateralis muscle using peripheral quantitative computed tomography (pQCT), and vastus lateralis muscle biopsies were obtained 1week prior to and 72h following the last training bout. Quantification of myofibril and mitochondrial areas in type I (positive for MyHC I) and II (positive for MyHC IIa/IIx) fibers was performed using immunohistochemistry (IHC) techniques. Relative myosin heavy chain and actin protein abundances per wet muscle weight as well as citrate synthase (CS) activity assays were also obtained on tissue lysates. Training increased whole-body lean mass, mid-thigh muscle cross-sectional area, mean and type II fiber cross-sectional areas (fCSA), and maximal strength values for leg press, bench press, and deadlift (*p*<0.05). The intracellular area occupied by myofibrils in type I or II fibers was not altered with training, suggesting a proportional expansion of myofibrils with fCSA increases. However, our histological analysis was unable to differentiate whether increases in myofibril number or girth occurred. Relative myosin heavy chain and actin protein abundances also did not change with training. IHC indicated training increased mitochondrial areas in both fiber types (*p*=0.018), albeit CS activity levels remained unaltered with training suggesting a discordance between these assays. Interestingly, although pQCT-derived muscle density increased with training (*p*=0.036), suggestive of myofibril packing, a positive association existed between training-induced changes in this metric and changes in mean fiber myofibril area (*r*=0.600, *p*=0.018). To summarize, our data imply that shorter-term resistance training promotes a proportional expansion of the area occupied by myofibrils and a disproportional expansion of the area occupied by mitochondria in type I and II fibers. Additionally, IHC and biochemical techniques should be viewed independently from one another given the lack of agreement between the variables assessed herein. Finally, the pQCT may be a viable tool to non-invasively track morphological changes (specifically myofibril density) in muscle tissue.

## Introduction

Resistance training increases markers of muscle hypertrophy and functional strength (reviewed in Haun et al., 2019b). It is generally recognized that type I, type II, and mean muscle fiber cross-sectional areas (fCSA) increase with weeks to months of resistance training. However, the morphological adaptations that occur in muscle fibers during resistance training have not been fully elucidated (Jorgenson et al., 2020; Reggiani and Schiaffino, 2020). Researchers have utilized elegant techniques to show that the intracellular environment during muscle fiber hypertrophy can adapt in one of three ways (Roberts et al., 2020a). Conventional hypertrophy is the notion that fCSA increases involve the proportional expansion of myofibrils and the non-myofibril components of the muscle fiber (Jorgenson et al., 2020), and there are reports to support this hypothesis (Claassen et al., 1989; Shoepe et al., 2003; Roberts et al., 2018). Myofibril packing is the notion that resistance training leads to a disproportionate increase in space occupied by myofibrils within the muscle fiber, and this acts to push the cell outward thereby facilitating fCSA increases (Phillips, 2000). Lastly, there is evidence to suggest that the expansion of the sarcoplasm predominates during muscle fiber hypertrophy (MacDougall et al., 1982; Haun et al., 2019a), and this has been termed sarcoplasmic hypertrophy (Roberts et al., 2020a). As a contextual example, consider an individual who possesses muscle fibers with 80% of the intracellular area occupied by myofibrils. If this individual experiences muscle fiber hypertrophy with resistance training, post-training intracellular myofibril areas could either be: (a) maintained ~80% with conventional hypertrophy, (b) increased to ~90% with myofibril packing, or (c) reduced to ~70% with sarcoplasmic hypertrophy. Visual depictions of these processes are provided in recent reviews (Jorgenson et al., 2020; Roberts et al., 2020a). Critically, these theses are predicated on total intracellular space occupied by myofibrils and do not consider if hypertrophy coincides with an increase in myofibril number or an increase in the girth of resident myofibrils. To this end, this issue remains unresolved in the field.

There are also conflicting reports regarding how mitochondrial volume density is affected by resistance training (Parry et al., 2020). In this regard, some studies suggest mitochondrial volume density increases in proportion with hypertrophy (Tesch et al., 1990; Salvadego et al., 2013; Porter et al., 2015), while other studies have reported that a dilution in mitochondrial volume density occurs with training (MacDougall et al., 1979, 1982; Luthi et al., 1986; Tesch et al., 1987; Haun et al., 2019a). Additionally, a surrogate marker of mitochondrial volume density (citrate synthase (CS) activity) has been reported to increase following 12weeks of resistance training, suggesting increases in mitochondrial volume density occurred more rapidly than increases in muscle fiber growth (Tang et al., 2006). While these data are conflicting, we have posited that the expansion of mitochondria is likely needed in muscle fibers that grow in response to resistance training in order to energetically support anabolic processes (e.g., increases in myofibril protein synthesis; Roberts et al., 2018).

Another unexplored area of exercise physiology is how size changes in myonuclear domains with resistance training are related to intracellular morphology. A central tenet of the myonuclear domain theory is that myonuclear accretion is needed in a growing muscle fiber in order to maintain the biosynthetic processes of intracellular organelles (Allen et al., 1999). To this end, we have hypothesized that if muscle fiber or myonuclear domain sizes became too large with resistance training, this might elicit “transcriptional burden” in resident myonuclei and lead to a disproportionate expansion of non-contractile components (Roberts et al., 2020a). These hypotheses are partially supported by multiple studies that have shown resistance training to increase myonuclear accretion in order to maintain a pseudo-rigid myonuclear domain size (Conceicao et al., 2018). However, no study has attempted to associate resistance training-induced changes in myonuclear domain sizes with changes in intracellular myofibril density or mitochondrial volume density.

In lieu of the disparate findings and knowledge gaps presented above, the purpose of this study was to use phalloidin-actin staining and mitochondrial immunohistochemical staining (*via* mitochondrial import receptor subunit TOM20 homolog (TOMM20) antibody) to examine how 10weeks of resistance training affects areas occupied by myofibrils and mitochondria in type I (MyHC I-positive) and II (MyHC IIa/IIx-positive) muscle fibers. Additionally, we sought to determine whether training-induced changes in myonuclear domain sizes in type I or type II fibers were associated with changes in myofibril and mitochondrial areas. As an exploratory analysis, biochemical methods that have been used to indirectly assess myofibril and mitochondrial densities were also performed to examine: (i) whether these variables were altered with training and (ii) whether data yielded from these techniques were associated with data generated through phalloidin-actin staining and TOMM20 immunohistochemistry (IHC). Finally, as an additional exploratory analysis, the radiological density of the vastus lateralis muscle was obtained prior to and following training using a peripheral quantitative computed tomography (pQCT) scanner and compared to data yielded from the aforementioned techniques to see whether significant associations existed. This analysis was motivated by prior studies suggesting that the radiological density of muscle tissue largely represents myofibril protein density (Claassen et al., 1989; Rasch et al., 2007; Lamb et al., 2020). We hypothesized that areas occupied by myofibrils and mitochondria in both fiber types would decrease relative to changes in fiber area (i.e., type I and II fibers would demonstrate sarcoplasmic hypertrophy). Our rationale for this hypothesis was our own laboratory’s observations that these processes occur (Haun et al., 2019a). Moreover, we hypothesized that there would be negative associations between training-induced changes in myonuclear domain sizes with changes myofibril and mitochondrial areas. For our exploratory analysis, we hypothesized that indirect measures of myofibril density and mitochondrial volume density (i.e., relative myosin heavy chain abundance and CS activity levels) would not be significantly associated with variables yielded from our histological assessments. Additionally, we hypothesized that training-induced changes in pQCT-determined muscle density would not be significantly associated with changes in muscle fiber myofibril areas.

## Materials and Methods

### Ethics Approval and Participant Inclusion Criteria

All procedures were approved by the Institutional Review Board at Auburn University (Protocol #20-136 MR 2004), and this study conformed to the standards set by the latest revision of the Declaration of Helsinki. Eligible participants had to be male, between the ages of 18–30years, free from cardio-metabolic diseases (e.g., morbid obesity, type II diabetes, severe hypertension), and free from medical conditions that precluded the collection of muscle biopsies. Additionally, participants could not be current smokers and could not have recently engaged in full-body resistance training >1day per week. Eligibility criteria indicated that participants could not have not consumed supplemental protein, creatine, or agents that affect anabolic hormones (testosterone boosters, growth hormone boosters, etc.) 2months prior to the training intervention. Additionally, participants were instructed that they could not self-administer nutritional supplements or consume other potentially ergogenic agents (e.g., pre-workout beverages) while enrolled in the study. Interested participants provided verbal and written consent to participate prior to data collection procedures outlined below.

### Study Design

**Figure 1:**
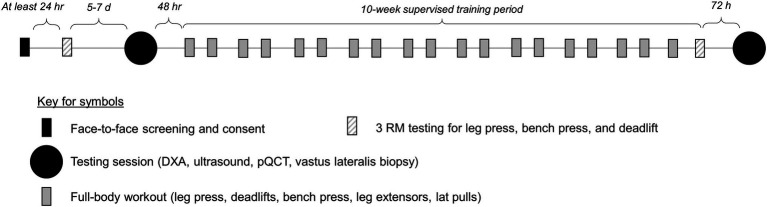
Study design. This figure illustrates the study design.

The following methods sections provide descriptions of strength testing, testing sessions, and resistance training. A summary schematic of the study design is presented in Figure 1.

### 3RM Strength Testing

Three repetition maximum (3RM) strength testing for the leg press, bench press, and deadlift exercises occurred twice in the study, and obtained values were used to estimate 1RM values according to methods provided by the National Strength and Conditioning Association (Haff and Triplett, 2016). We opted to implement 3RM testing versus 1RM testing given that we have found multiple RM testing to yield more a more representative depiction of strength for given exercises in untrained individuals (*unpublished observations*). Moreover, others have reported that up to 5RM testing yields highly reliable data regarding true 1RM strength versus 10RM or 20 RM tests (Reynolds et al., 2006). The first 3RM session occurred 5–7days prior to the PRE testing session, and the second 3RM session occurred as the last workout for the 10-week training period (which was 72h prior to the POST testing session). 3RM testing procedures were identical to procedures that we have previously published (Mobley et al., 2017a). Participants were also familiarized with the training during testing of 3RMs. All testing occurred under direct supervision of a technician holding the Certified Strength and Conditioning Specialist Certification from the National Strength and Conditioning Association.

### Pre and Post Testing Sessions

Testing sessions occurred during the morning hours (06:00–11:00) following an overnight fast. These sessions occurred 3–5days prior to the 10-week training intervention (PRE) and 72h following the last training bout (POST). During these sessions, participants reported to the Auburn University School of Kinesiology wearing casual sports attire (i.e., athletic shirt and shorts, tennis shoes). Additionally, participants were given four-day food logs prior to the PRE and POST testing sessions, and we requested that they return these logs during the visits after having completed the log over two typical weekdays and weekend days. PRE and POST four-day food logs were analyzed using commercially available software (ESHA Food Processor v.11.1, ESHA Research; Salem, OR, United States). Calorie and protein intake data are presented as daily values, which were averaged over the 4-day entries.

#### Body Composition Assessments

Participants submitted a urine sample (~5ml) to assess urine-specific gravity levels using a handheld refractometer (ATAGO; Bellevue, WA, United States). Notably, all participants possessed values less than 1.020 indicating that they were well-hydrated (American College of Sports et al., 2007). Height and body mass were then assessed using a digital column scale (Seca 769; Hanover, MD, United States). Participants then underwent a full-body dual-energy X-ray absorptiometry (DXA) scan (Lunar Prodigy; GE Corporation, Fairfield, CT, United States) for determination of whole-body lean soft tissue mass (LSTM). Notably, participants laid supine on the scanning table 5min prior to the initiation of the scan. Quality assurance testing and calibration were performed in the morning of data collection days to ensure the scanner was operating to manufacturer specification. Scans were analyzed by the same technician using the manufacturer’s standardized software. Twenty-four-hour test-retest reliability using intraclass correlation_3,1_ (ICC_3,1_), standard error of the measurement (SEM), and minimal difference to be considered real (MD) was previously determined for LSTM and resulted in ICC_3,1_, SEM, and MD values of 0.99, 0.36, and 0.99kg, respectively.

Following the DXA scan, a cross-sectional image of the right thigh at 50% of the femur length was acquired using a pQCT scanner (Stratec XCT 3000, Stratec Medical, Pforzheim, Germany). Scans were acquired using a single 2.4mm slice thickness, a voxel size of 0.4mm, and scanning speed of 20mm/s. All images were analyzed for mid-thigh muscle cross-sectional area (mCSA, cm^2^) and vastus lateralis radiological muscle density (mg/cm^3^) using the pQCT BoneJ plugin freely available through ImageJ analysis software (NIH, Bethesda, MD). All scans were performed and analyzed by the same investigator. Twenty-four-hour test-retest reliability ICC_3,1_, SEM, and MD was previously determined for mCSA and resulted in ICC_3,1_, SEM, and MD values of 0.99, 0.84, and 2.32cm^2^, respectively. Notably, only skeletal muscle tissue within the vastus lateralis region was analyzed for tissue density, as intermuscular and subcutaneous fat was excluded.

#### Muscle Biopsies

After body composition and mid-thigh assessments, skeletal muscle biopsies were obtained from the right thigh (i.e., vastus lateralis; in the same plane as ultrasound and pQCT assessments) using a 5-gauge needle with suction and sterile procedures. Briefly, participants were instructed to lie in a supine position on an athletic training table. Lidocaine (1%, 1.5ml) was injected subcutaneously above the skeletal muscle fascia at the collection site. After 5min of allowing the anesthetic to take effect, a small pilot incision was made using a sterile Surgical Blade No. 11 (AD Surgical; Sunnyvale, CA, United States), and the biopsy needle was inserted into the pilot incision just below the fascia. Approximately 50–80mg of skeletal muscle was removed using a 5-gauge Bergstrom biopsy needle (Millennium Surgical Corp.; Narberth, PA, United States) and applied suction (Evans et al., 1982). Following biopsies, tissue was rapidly teased of blood and connective tissue. A portion of the tissue (~10–20mg) was preserved in freezing media for histology (Tissue-Tek^®^, Sakura Finetek Inc.; Torrance, CA, United States), slow frozen in liquid nitrogen-cooled isopentane, and subsequently stored at −80°C. Another portion of the tissue (~30–50mg) was placed in pre-labeled foils, flash frozen in liquid nitrogen, and subsequently stored at −80°C until the isolation of myofibrils and other assays described below.

### Resistance Training

Two days following the PRE testing battery, resistance training commenced. Training occurred 2days per week where participants were allowed to train from either 07:00–09:00 or 15:30–18:30 on Monday or Tuesday and Wednesday or Thursday of each week. Participants were instructed to not perform other vigorous exercise activities outside of the study.

Participants performed three lower body exercises to target the quadriceps muscles and two accompanying compound upper body exercises. Prior to beginning each training session, participants were instructed to perform a general warm-up involving 25 jumping jacks, 10 bodyweight squats, and 10 push-ups for two rounds. Afterward, participants engaged in exercises that included the leg press, barbell deadlift, bilateral leg extensions, bench press, and a machine pulldown exercise designed to target the elbow flexors and latissimus dorsi muscles. Two warm-up sets were allowed which were <50% of the working set. Thereafter, working sets were performed according to the set/repetition scheme shown in Table 1. Participants were recommended to take 2min of rest between each exercise set. However, participants were allowed to proceed to the next exercise without 2min of rest if they felt prepared to execute exercises with appropriate technique. Additionally, if participants desired more than 2min of rest, this was allowed.

**Table 1:** Resistance training scheme.

Week	Day	Set × rep scheme	% initial 1RM
1	M/T	4×10	50
	W/R	5×6	56
2	M/T	4×10	55
	W/R	5×6	65
3	M/T	4×10	60
	W/R	5×6	74
4	M/T	4×10	65
	W/R	5×6	84
5^*^	M/T	4×10	50
	W/R	5×6	50
6	M/T	4×10	65
	W/R	5×6	84
7	M/T	4×10	70
	W/R	5×6	90
8	M/T	4×10	75
	W/R	5×6	96
9	M/T	4×10	80
	W/R	5×6	98
10	M/T	5×6	102
	W/R	Post strength test	---

The set x rep scheme presented above was for five exercises per workout (leg press, bench press, deadlift, leg extension, cable pulldown). Additionally, participants worked out either Monday or Tuesday and Wednesday or Thursday for two workouts per week. *, indicates deload week.

Training load for each exercise was progressed in a pre-programmed manner according to Table 1. However, participant feedback was also taken into consideration. In this regard, a degree of difficulty (RPE) scale was used during workouts where 0=“very easy” and 10=“very hard.” After each set for each exercise, RPE was gauged by training staff. If RPE was less than 6, then ~10% additional load was added to the exercise. If RPE was less than 4, then ~20% additional load was added to the exercise. If a set was not completed or RPE was 10, then load was adjusted according to the discretion of the research staff. All training occurred under direct supervision of research staff, and all volume was logged. All participants were instructed to maintain their normal dietary patterns throughout training as best as possible.

### Analyses of Muscle Specimens

*Isolation of myofibrillar and sarcoplasmic protein fractions.* Myofibrillar and sarcoplasmic protein isolations were performed based on our published “MIST” method (Roberts et al., 2020b), which was derived from Goldberg’s laboratory (Cohen et al., 2009). Tissue foils were removed from −80°C, and tissue was crushed on a liquid nitrogen-cooled ceramic mortar with a ceramic pestle. Powdered tissue (~20mg) was placed in 1.7-ml tubes pre-filled with ice-cold buffer (300μl; Buffer 1: 25mM Tris, pH 7.2, 0.5% Triton X-100, protease inhibitors) and weighed using an analytical scale sensitive to 0.0001g. Samples were homogenized using tight-fitting pestles and centrifuged at 1,500×g for 10min at 4°C. Supernatants (sarcoplasmic fraction) were collected and placed in new 1.7-ml microtubes on ice. As a wash step, the resultant myofibrillar pellet was resuspended in 300μl of Buffer 1 and centrifuged at 1,500×g for 10min at 4°C. The supernatant was discarded, and the myofibrillar pellet was solubilized in 400μl of ice-cold resuspension buffer (20mM Tris-HCl, pH 7.2, 100mM KCl, 20% glycerol, 1mM DTT, 50mM spermidine, protease inhibitors). On the same day of isolations, sarcoplasmic protein samples were assayed in duplicate for total protein using a commercial BCA assay protocol (Thermo Fisher Scientific, Waltham, MA, United States). The average coefficient of variation (CV) for duplicate readings was 2.3%, and sarcoplasmic protein concentrations were normalized to input muscle weights. Sarcoplasmic protein samples were then stored at −80°C until the CS activity assay described below. On the same day of isolations, SDS-PAGE myofibril preps were also made using 10μl resuspended myofibrils, 65μl distilled water (diH_2_O), and 25μl 4x Laemmli buffer. These preps were boiled for 5min and stored at −80°C until electrophoresis was described below.

#### Electrophoretic Determination of Myosin and Actin Content

The electrophoretic determination of relative myosin and actin content was performed as previously described by our laboratory (Roberts et al., 2018; Vann et al., 2020a,b) and others (Cohen et al., 2009). Briefly, SDS-PAGE preps (5μl) were loaded in duplicate on pre-casted gradient (4–15%) SDS-polyacrylamide gels (Bio-Rad Laboratories, Hercules, CA, United States) and subjected to electrophoresis at 200V for 40min using pre-made 1x SDS-PAGE running buffer (VWR International, Radnor, PA, United States). Following electrophoresis gels were rinsed in diH_2_O for 10min and immersed in Coomassie stain (LabSafe GEL Blue; G-Biosciences; St. Louis, MO, United States) for 1h. Thereafter, gels were destained in diH_2_O for 2h, bright field imaged using a gel documentation system (ChemiDoc; Bio-Rad Laboratories), and band densitometry was performed with a gel documentation system and associated software. Given that a standardized volume from all samples was loaded onto gels, myosin and actin band densities were normalized to input muscle weights for relative expression. Our laboratory has reported that this method yields exceptional sensitivity in detecting 5–25% increases in actin and myosin content (Roberts et al., 2018). Notably, actin and myosin content were the only two myofibrillar protein targets of interest given that the combination of these proteins makes up a majority (~70%) of the myofibrillar protein pool as determined *via* proteomics (Vann et al., 2020b). The average coefficient of variation values for all duplicates was 2.3% for myosin heavy chain band densities and 2.5% for actin band densities.

#### CS Activity Assay

Sarcoplasmic fractions were batch process-assayed for total protein content using a BCA Protein Assay Kit (Thermo Fisher Scientific; Waltham, MA, United States). Thereafter, samples were batch processed for CS activity as previously described (Roberts et al., 2018). This marker was used as a surrogate for mitochondrial volume density according to previous literature suggesting CS activity highly correlates with electron micrograph images of mitochondrial content in skeletal muscle (*r*=0.84, *p*<0.001; Larsen et al., 2012), and recent literature showing this marker increases in spinal cord patients with evoked resistance training (Gorgey et al., 2020). The assay utilized is based on the reduction in 5, 50-dithiobis (2-nitrobenzoic acid; DTNB) at 412nm (extinction coefficient 13.6mmol/l/cm) coupled to the reduction in acetyl-CoA by the CS reaction in the presence of oxaloacetate. Briefly, 12.5μg of whole-tissue sample lysates was added to a mixture composed of 0.125mol/l Tris-HCl (pH 8.0), 0.03mmol/l acetyl-CoA, and 0.1mmol/l DTNB. The reaction was initiated by the addition of 5μl of 50mM oxaloacetate, and the absorbance change was recorded for 1min using a microplate spectrophotometer (BioTek; Winooski, VT, United States). The average coefficient of variation values for all duplicates was less than 10%.

#### IHC for Determining Fiber Type, Phalloidin-Actin Staining, and TOMM20 IHC

Sections from OCT-preserved samples were sectioned at a thickness of 10μm using a cryotome (Leica Biosystems; Buffalo Grove, IL, United States) and adhered to positively charged histology slides. Slides were then stored at −80°C until batch processing occurred for procedures described below.

For type I and II fCSA determination, sections were assayed as previously described by our laboratory (Vann et al., 2020a). Notably, only type I and II fibers were quantified, rather than I/IIa/IIx, given that: (a) less than ~5% of total fibers are typically IIx from samples we have collected (unpublished observations), and (b) our fluorescent microscope only contains three detection filters (DAPI, FITC, TRITC). Standardized measurements of type I, type II, and mean fCSAs were performed using open-sourced software (MyoVision; Wen et al., 2018). A pixel conversion ratio value of 0.964μm/pixel was used to account for the size and bit depth of images, and a detection range of detection from 500 to 12,000μm^2^ was used to ensure artifact was removed (e.g., large fibers which may have not been in transverse orientation or structures between dystrophin stains which were likely small vessels).

**Figure 2:**
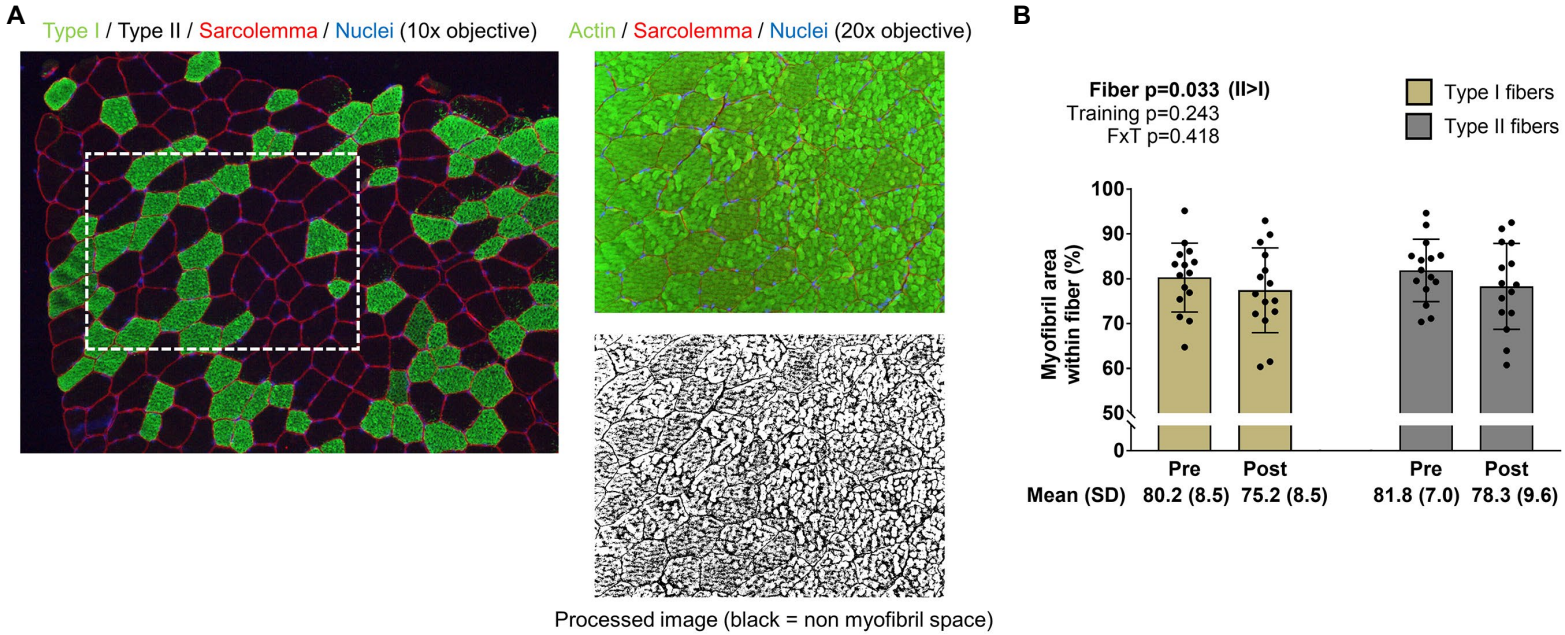
Type I and II fiber myofibril areas with training. The figure in panel **A** is from IHC and phalloidin-actin staining; specifically, this figure demonstrates how myofibril areas were quantified using 20x images and cross-referenced to fiber type from 10x images of serial sections. Panel **B** shows how training affected myofibril areas in type I and II fibers. Bar graph data are presented as means ± standard deviation values, and individual participant data are overlaid. *n*=15 participants. Abbreviation: FxT, fiber x training interaction.

For the determination of myofibril area per fiber, F-actin labeling using Alexa Fluor 488-conjugated (AF488) phalloidin was performed according to previous reports (Gokhin et al., 2008; Duddy et al., 2015; Haun et al., 2019a). This staining method allowed the identification of the sarcolemma (Texas Red filter), myofibrils (FITC filter), and myonuclei (DAPI filter). ImageJ (NIH) was used to quantify myofibril area per fiber. Briefly, images were split into RGB channels, and the green channel image was converted to grayscale. The threshold function in ImageJ was then used to generate binary black/white images of stained versus unstained portions of fibers. Thereafter, fibers were manually traced using the polygon function, and myofibril areas were provided as a percentage per fiber area. A visual representation of this image analysis is provided in Figure 2 in the results section. Fibers that were quantified in this regard were manually matched to fibers on 10x images to derive myofibril areas for type I and type II fibers. Myonuclei from 20x images were also manually assigned to fibers to extrapolate myofibril-myonuclei relationships. Notably, our histological analysis was unable to differentiate whether increases in myofibril number or increases in the girth of resident myofibrils occurred.

**Figure 3:**
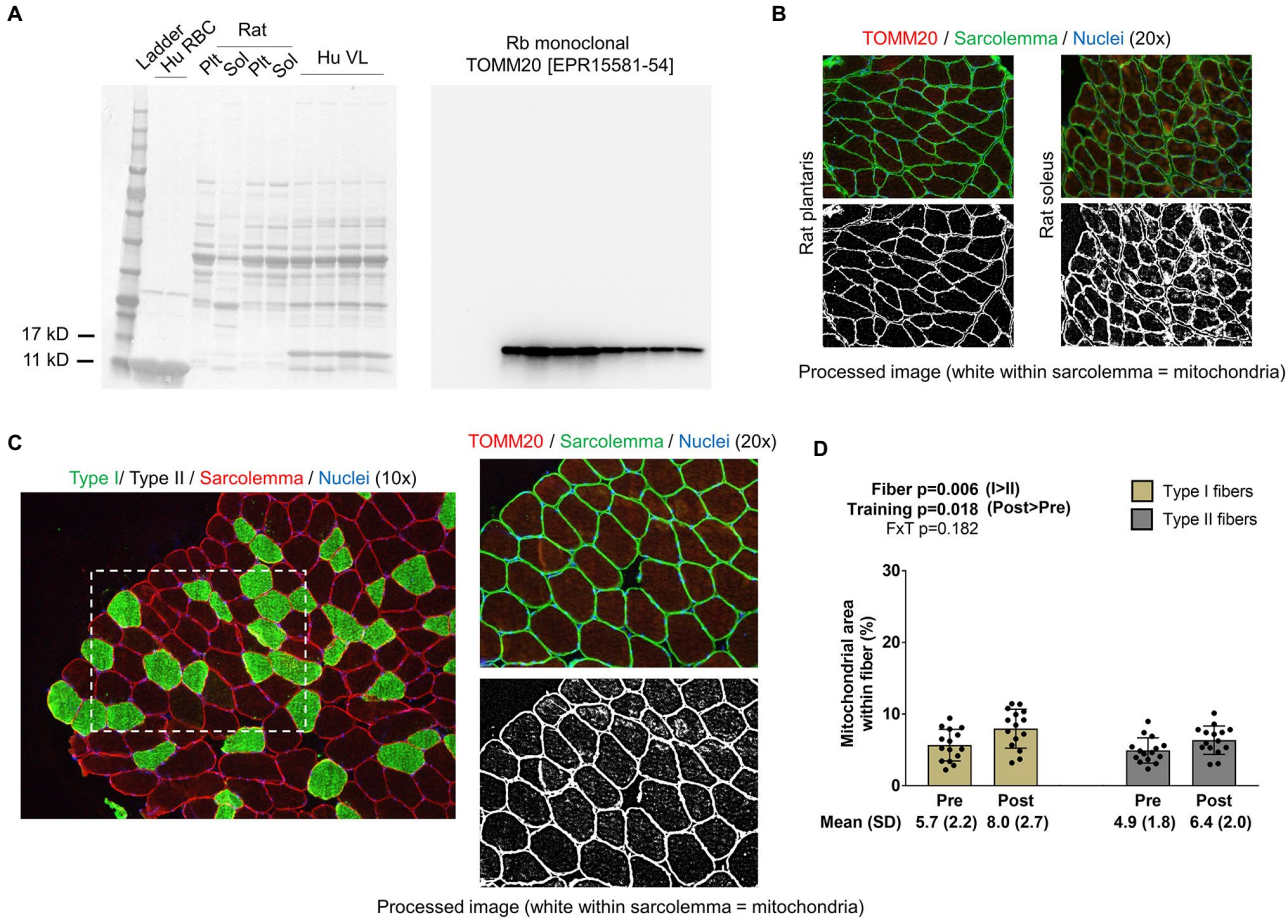
Type I and II fiber mitochondrial areas with training. Panel **A** shows Western blotting validation results regarding the veracity of the TOMM20 antibody. The left inset is the Ponceau stain of human red blood cells (Hu RBC), rat plantaris (Plt) and soleus (Sol) lysates, and human vastus lateralis muscle (Hu VL). The right inset contains results from Western blotting. Panel **B** shows IHC validation results from rat Plt and Sol muscles (20x images). Panel **C** shows IHC and TOMM20 staining from our human participants; specifically, this figure demonstrates how mitochondrial areas were quantified using 20x images and cross-referenced to fiber type from 10x images of serial sections. Panel **D** shows how training affected mitochondrial areas in type I and II fibers. Bar graph data are presented as means ± standard deviation values, and individual participant data are overlaid. *n*=15 participants. Abbreviation: FxT, fiber x training interaction.

For the determination of mitochondrial area per fiber, TOMM20 IHC was performed in accordance with a previous report that used TOMM20 IHC to estimate mitochondrial volume density in colorectal tissue (Kaldma et al., 2014). Briefly, a second set of serial sections was air-dried for 10min. Sections were then washed in PBS for 5min and permeabilized for 20min using 0.5% Triton X in PBS. Sections were then blocked with 100% Pierce Super Blocker (Thermo Fisher Scientific) for 25min and subsequently incubated for 60min in a primary antibody solution containing: (i) a base buffer of 1x PBS, (ii) 5% Pierce Super Blocker, (iii) 2.5% (or 1:50 dilution) of a commercially available rabbit anti-TOMM20 monoclonal IgG antibody (catalog #: ab232589; Abcam; Cambridge, MA, United States), and (iv) 2.5% (or 1:50 dilution) of a commercially available mouse anti-dystrophin IgG1 antibody (MANDRA1 supernatant; Developmental Studies Hybridoma Bank). Sections were then washed for 5min with PBS and incubated in the dark for 60min with a secondary antibody solution containing: (i) a base buffer of 1x PBS, (ii) 5% Pierce Super Blocker, and (iii) Texas Red-conjugated anti-rabbit IgG (catalog #: TI-1000; Vector Laboratories) and Alexa Fluor 488-conjugated anti-mouse IgG1 (catalog #: A-11001; Thermo Fisher Scientific; ~6.6μl of all secondary antibodies per 1ml of blocking solution, or a 1:150 dilution). Sections were washed for 5min in PBS, air-dried, and mounted with fluorescent media containing DAPI (catalog #: GTX16206; GeneTex Inc.). Following mounting, digital images were immediately captured with a fluorescent microscope (Nikon Instruments) using the 20x objective. Exposure times were 200 milliseconds for FITC and TRITC and 10ms for DAPI imaging. This staining method allowed the identification of the sarcolemma (FITC filter), mitochondria (Texas Red filter), and myonuclei (DAPI filter). ImageJ (NIH) was used to quantify mitochondrial area per fiber as described above for phalloidin-actin staining. Similar to phalloidin images, TOMM20 images were split into RGB channels, and the red channel image was converted to grayscale. The threshold function in ImageJ was then used to generate binary black/white images of stained versus unstained portions of fibers. Thereafter, fibers were manually traced using the polygon function, and mitochondrial areas were provided as a percentage per fiber area. A visual representation of this image analysis is provided in Figure 3 in the results section. Fibers that were quantified in this regard were manually matched to fibers on 10x images to derive myofibril areas for type I and type II fibers. Myonuclei from 20x images were also manually assigned to fibers to extrapolate mitochondrial-myonuclei relationships.

Notably, we extensively validated the TOMM20 antibody by running Western blots on: (i) muscle tissue lysates obtained in the current study, (ii) red blood cell lysates (note, red blood cells lack mitochondria making this a negative control), (iii) rat plantaris muscle, and (iv) rat soleus muscle. Additionally, we performed TOMM20 IHC on rat soleus and plantaris muscle. Assayed rat muscle was banked from a prior study published from our laboratory, where tissue collection was approved by the Auburn University Institutional Animal Care and Use Committee (Mobley et al., 2017b). Outcomes from these validation studies are presented in the results section.

### Statistics

Statistical analyses were performed using SPSS (version 25; IBM SPSS Statistics Software, Chicago, IL, United States). The only dependent variables to be analyzed using two-way ANOVAs were fiber type-specific changes in myofibril and mitochondrial areas (fiber: type I versus type II x time: PRE versus POST). If significant fiber type x training interactions were observed for either of these two variables, it was decided *a priori* that the model would be decomposed using dependent samples t tests to examine PRE versus POST for each fiber type and independent samples t tests to compare each fiber type at PRE and POST. PRE to POST changes in other dependent variables were analyzed using dependent samples t tests. Select associations between dependent variables were also analyzed using Pearson correlations. All data in tables and figures are presented as mean±standard deviation (SD) values, and statistical significance was set at *p*<0.05.

## Results

### General Training Adaptations

The 15 participants were 24±4years and possessed a body mass index of 23.9±3.1kg/m^2^. Most participants had a workout compliance of 100%, except three, whose compliance rate was 90% (or two missed workouts). Table 2 contains general training adaptations for the 15 participants. Training significantly increased whole-body LSTM according to DXA, mid-thigh muscle cross-sectional area according to pQCT, mean and type II fCSA according to 10x IHC images, type II fiber myonuclear domain size to 10x IHC images, estimated 1RM leg press, and estimated 1RM deadlift. Training did not increase type I fCSA or type I fiber myonuclear domain size according to 10x IHC images. Regarding 10x IHC data, total numbers of fibers quantified were 56±13 type I and 92±35 type II fibers at PRE and 48±19 type I and 81±25 type II fibers at POST.

**Table 2:** General training adaptations.

Variable (units)	Time	Values (*n*=15)	value of p
DXA LSTM (kg)	PRE	56.4 ± 6.7	<0.001
	POST	58.4 ± 6.8	
	% change	3.7 ± 3.1	
Mid-thigh mCSA (cm^2^)	PRE	145 ± 21	<0.001
	POST	162 ± 20	
	% change	11.9 ± 5.4	
Type I fCSA (μm^2^)	PRE	4,655 ± 1,112	0.13
	POST	5,092 ± 1,138	
	% change	14.6 ± 28.0	
Type I MND size (μm^2^)	PRE	2,367 ± 693	0.879
	POST	2,397 ± 508	
	% change	6.7 ± 28.9	
Type II fCSA (μm^2^)	PRE	5,410 ± 1,507	0.008
	POST	6,561 ± 1,520	
	% change	31.9 ± 36.7	
Type II MND size (μm^2^)	PRE	2,251 ± 697	0.043
	POST	2,672 ± 428	
	% change	27.3 ± 38.4	
Mean fCSA (μm^2^)	PRE	5,105 ± 1,291	0.013
	POST	6,024 ± 1,238	
	% change	26.5 ± 32.0	
Est. 1RM leg press (kg)	PRE	157 ± 54	<0.001
	POST	231 ± 71	
	% change	49.6 ± 17.3	
Est. 1RM deadlift (kg)	PRE	105 ± 23	<0.001
	POST	141 ± 24	
	% change	36.2 ± 12.6	

*All data presented as mean±standard deviation values. Abbreviations: DXA, dual-energy X-ray absorptiometry; LSTM, lean/soft tissue mass; mCSA, muscle cross-sectional area; fCSA, fiber cross-sectional area; MND, myonuclear domain; Est. 1RM, estimated one repetition maximum*.

Although self-reported dietary data were not a primary outcome of this investigation, they are provided here for reference. Participants reported consuming more calories per day at POST versus PRE (2,152±842kcal/d versus 1,606±526kcal/d, *p*=0.028) and more protein per day at POST versus PRE (111±43g/d versus 79±35g/d, *p*=0.016). Thus, the positive training adaptations (e.g., increases in whole-body LSTM, mid-thigh mCSA, and strength) may have been the combination of progressive overload as well as increases in calories and protein consumed per day.

### Changes in Type I and II Fiber Myofibril Areas

The image in Figure 2A demonstrates how myofibril areas in type I and II fibers were quantified as described in the methods section. Data in Figure 2B indicate that training did not significantly affect myofibril areas in type I or II fibers (training *p*=0.243, fiber type x training interaction *p*=0.418; *post hoc* analysis within fibers were not performed). However, there was a fiber effect, where myofibrils occupied more intracellular space in type II versus type I fibers (*p*=0.033). Regarding these 20x IHC data, 11±4 type I and 16±6 type II fibers were quantified at PRE, and 8±4 type I and 13±4 type II fibers were quantified at POST.

### Changes in Type I and II Muscle Fiber Mitochondrial Areas

Western blotting validation experiments for TOMM20 show the presence of one sole band ~15kD in all rat and human muscle samples and the absence of said band in red blood cell lysates (Figure 3A); notably, band densities were greater in rat soleus versus plantaris muscle lysates. Regarding IHC validation, rat soleus muscle fibers (which contain >90% type I fibers) visually present a higher enrichment of TOMM20 compared to rat plantaris muscle fibers (which contain >90% type I fibers; Figure 3B). The image in Figure 3C demonstrates how mitochondrial areas in type I and II fibers were quantified from our participants that resistance trained using image threshold and fiber tracing techniques. Data in Figure 3D show that no fiber x training interaction existed for mitochondrial areas (*p*=0.182). However, there was a fiber effect where mitochondria occupied more intracellular space in type I versus type II fibers (*p*=0.006). Additionally, there was a training effect where mitochondrial areas were greater at POST versus PRE (*p*=0.018). Regarding these 20x IHC data, 10±3 type I and 15±8 type II fibers were quantified at PRE, and 9±3 whole type I and 12±5 whole type II fibers were quantified at POST.

### Relationships Between Myonuclear Domain and fCSA Sizes Versus Myofibril Areas

**Figure 4:**
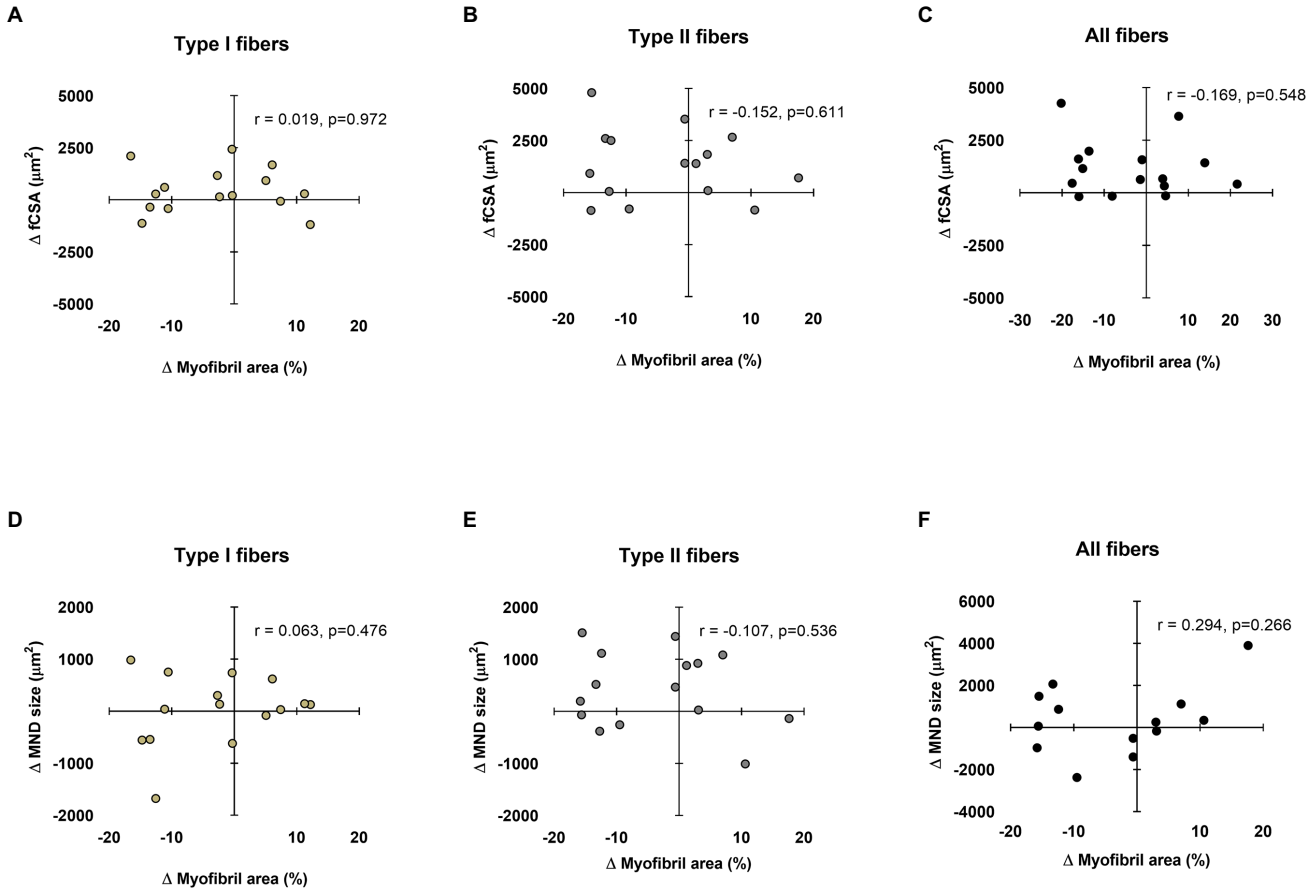
Relationships between myonuclear domain and fiber cross-sectional area (fCSA) sizes versus myofibril area. These plots demonstrate the relationships between changes in type I, II, and mean fCSA versus change in myofibril area (panels **A–C**, respectively), as well as the change in type I, II, and mean fiber myonuclear domain (MND) sizes versus change in myofibril area (panels **D–F**, respectively). n=13-15 participants in each panel.

Data in Figure 4 show associations between training-induced changes in myonuclear domain and fCSA values versus training-induced changes in type I and II fiber myofibril areas. No significant associations existed between the following variables: (i) change in type I fCSA from PRE to POST and change in type I fiber myofibril area from PRE to POST (Figure 4A), (ii) change in type II fCSA from PRE to POST and change in type II fiber myofibril area from PRE to POST (Figure 4B), or (iii) change in mean fCSA from PRE to POST and change in mean fiber myofibril area from PRE to POST (Figure 4C). Additionally, no significant associations existed between the following variables: (i) change in type I fiber myonuclear domain size from PRE to POST and change in type I fiber myofibril area from PRE to POST (Figure 4D), (ii) change in type II fiber myonuclear domain size from PRE to POST and change in type II fiber myofibril area from PRE to POST (Figure 4E), or (iii) change in mean fiber myonuclear domain size from PRE to POST and change in mean fiber myofibril area from PRE to POST (Figure 4F).

### Relationships Between Myonuclear Domain and fCSA Sizes Versus Mitochondrial Areas

**Figure 5:**
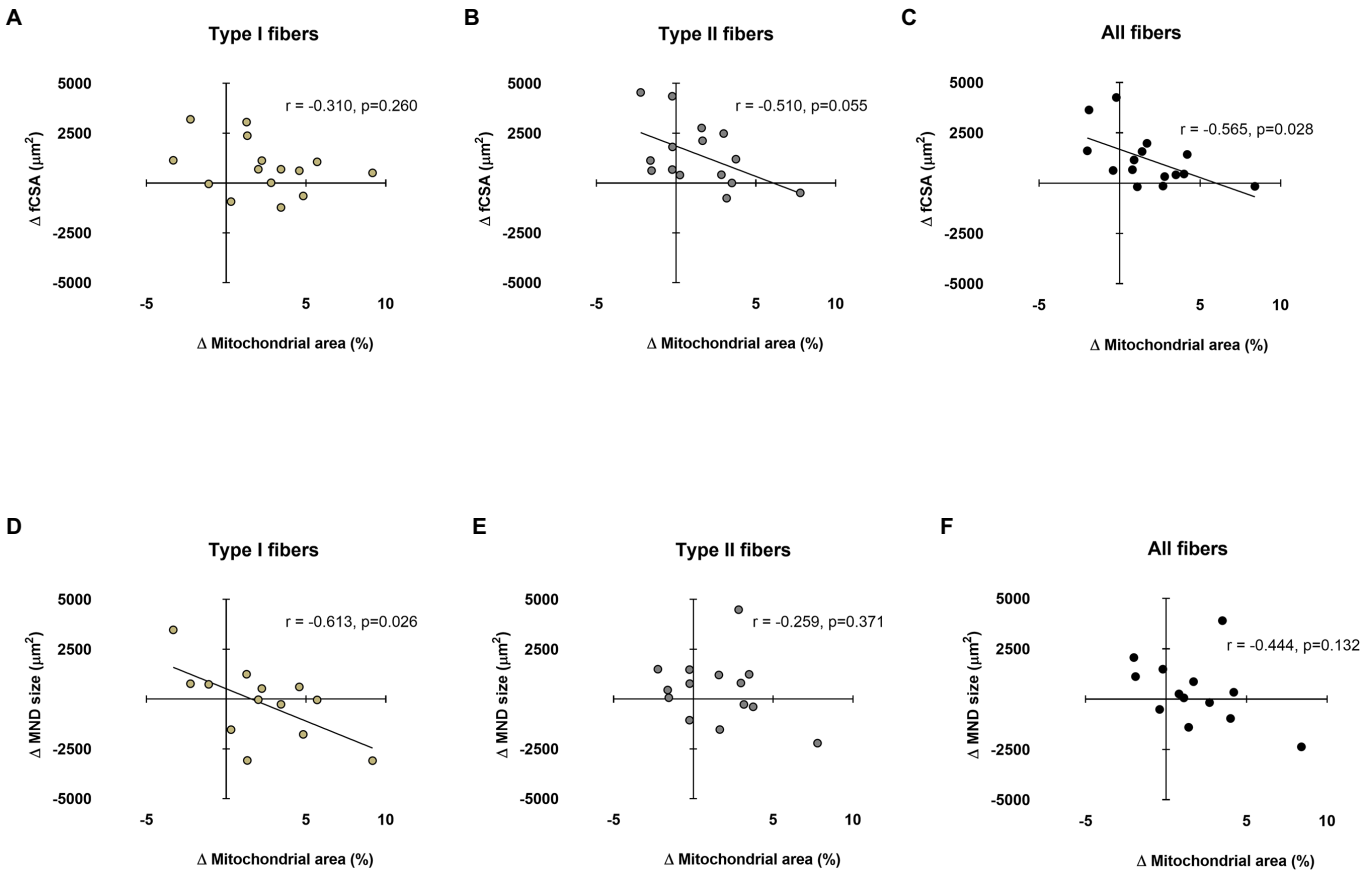
Relationships between myonuclear domain and fCSA sizes versus mitochondrial area. These plots demonstrate the relationships between changes in type I, II, and mean fCSA versus change in mitochondrial area (panels **A–C**, respectively) and the change in type I, II, and mean fiber myonuclear domain (MND) sizes versus change in mitochondrial area (panels **D–F**, respectively). n=13-15 participants in each panel.

Data in Figure 5 show associations between training-induced changes in myonuclear domain and fCSA values versus training-induced changes in type I and II fiber mitochondrial areas. No significant association existed between the change in type I fCSA from PRE to POST and change in type I fiber mitochondrial area from PRE to POST (Figure 5A). Moderate negative correlations existed for change in type II and mean fCSA from PRE to POST and change in type II and mean fiber mitochondrial area from PRE to POST (Figures 5B,C). A significant negative correlation also existed between the change type I fiber myonuclear domain size from PRE to POST and change in type I fiber mitochondrial area from PRE to POST (Figure 5D). No significant associations existed between the changes in type II or mean fiber myonuclear domain sizes from PRE to POST and changes in type II or mean fiber mitochondrial areas from PRE to POST (Figures 5E,F). Notably, two subjects were removed from data in panels d and f due to poor DAPI staining, and one subject was removed from panel e for the same reason.

### Radiological Density Data of the Vastus Lateralis

**Figure 6:**
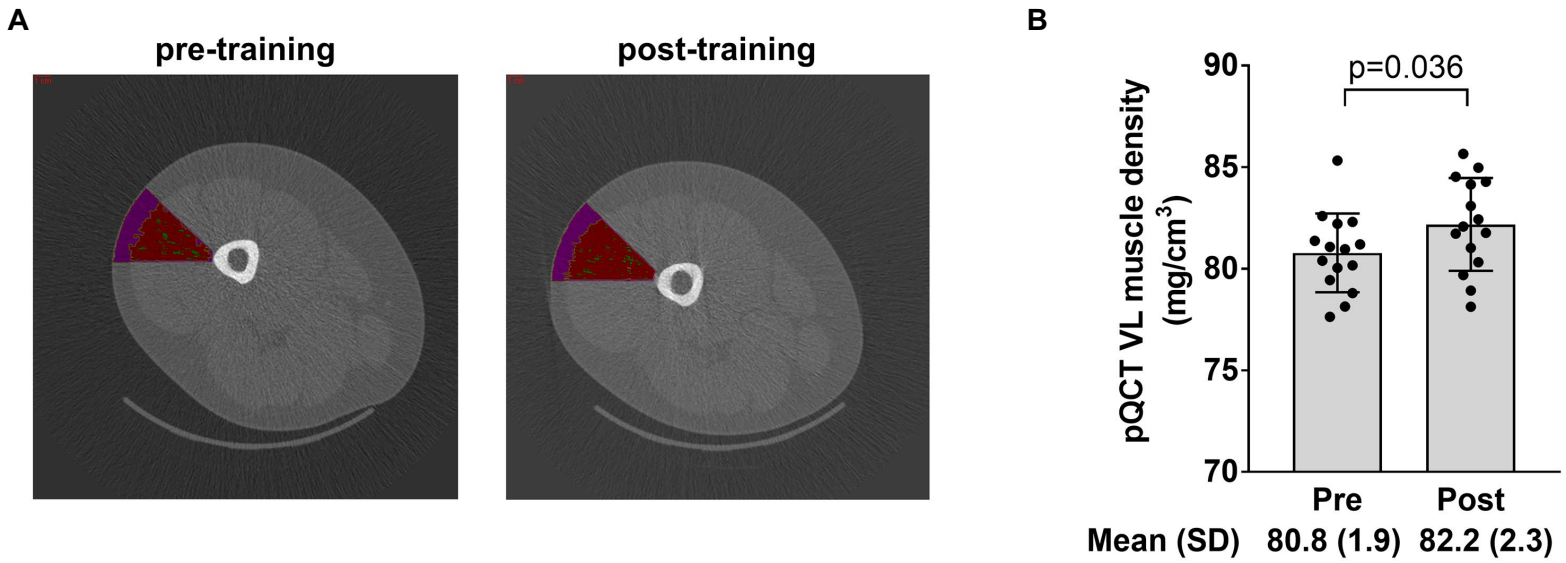
Radiological density data of the vastus lateralis. The figure in panel **A** demonstrates the region of interest drawn over the mid-thigh scan to extrapolate tissue density in a portion of the vastus lateralis (where the biopsy occurred) with the intent of correlating these data to vastus lateralis mean (type I and II) myofibril area. Panel **B** shows how training affected vastus lateralis (VL) density. Bar graph data are presented as means ± standard deviation (SD) values, and individual participant data are overlaid. *n*=15 participants.

The image in Figure 6A depicts the mid-thigh vastus lateralis region of interest that was quantified for muscle tissue density using the pQCT and associated analysis software. Data in Figure 6B show that training significantly increased muscle tissue density in this region (*p*=0.036).

### Electrophoresis and CS Activity Data

**Figure 7:**
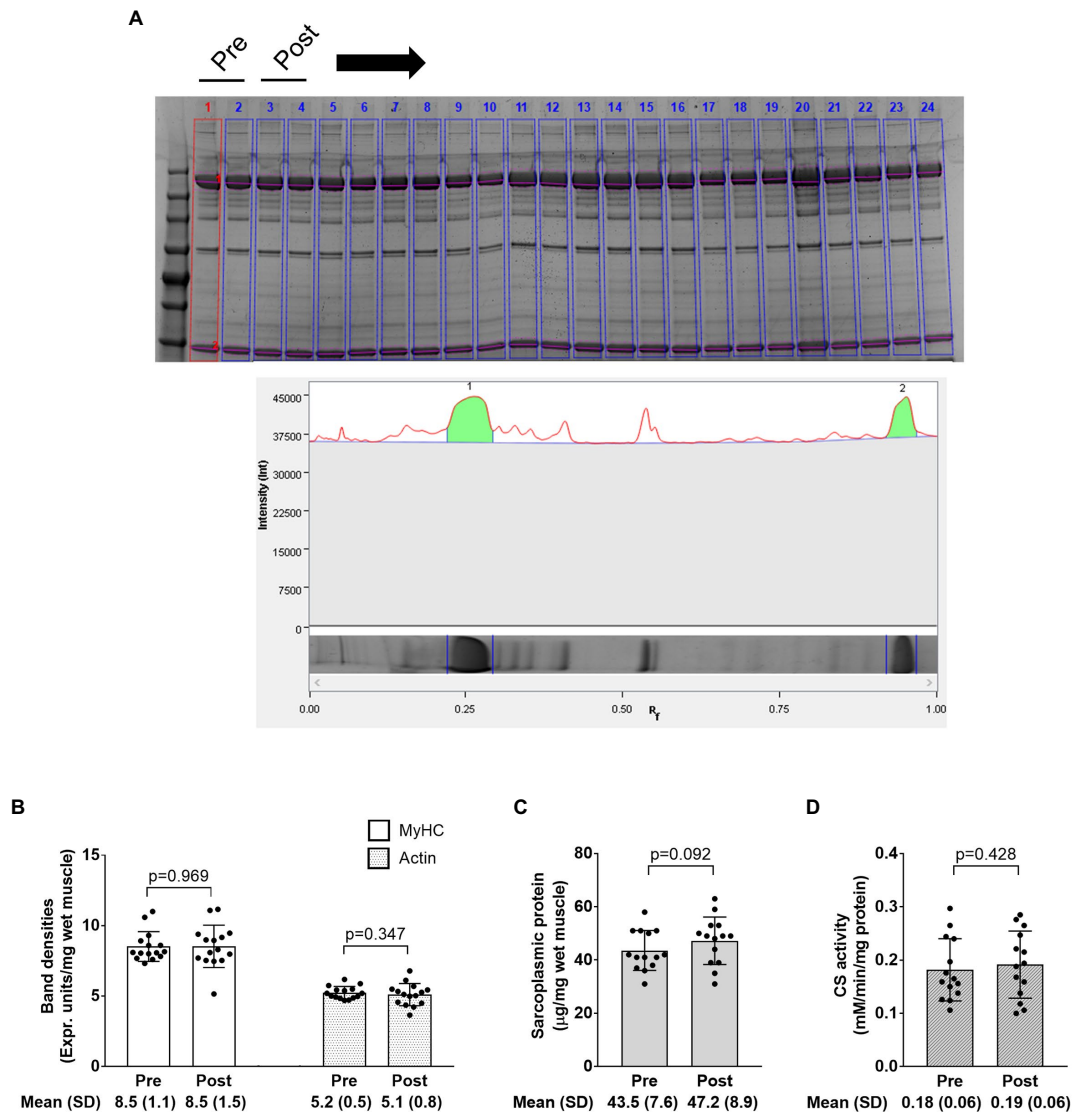
Electrophoresis, sarcoplasmic protein, and CS activity data. The figure in panel **A** is from SDS-PAGE; specifically, this figure demonstrates how the relative densities of myosin heavy chain and actin (lane 1 in this image) were quantified using band densitometry. Panel **B** shows how training affected relative myosin heavy chain (MyHC) and actin protein content per milligram wet muscle. Panel **C** shows how training affected sarcoplasmic protein concentrations per milligram wet muscle. Panel **D** shows how training affected CS activity levels, a surrogate of mitochondrial volume density. Bar graph data are presented as means ± standard deviation values, and individual participant data are overlaid. *n*=14 participants in the CS activity and sarcoplasmic protein panels, and *n*=15 participants in other panels.

The image in Figure 7A demonstrates how myosin heavy chain and actin bands were quantified as described in the methods section. Data in Figures 7B–D show that training did not significantly affect myosin heavy chain or actin band densities (*p*=0.969 and 0.347, respectively), sarcoplasmic protein concentrations (*p*=0.092), or CS activity (*p*=0.428). Notably, one subject was removed from data in panels c and d due to potential contamination of the sarcoplasmic protein pool.

### Associations Between Histological, Biochemical, and Radiological Techniques

**Figure 8:**
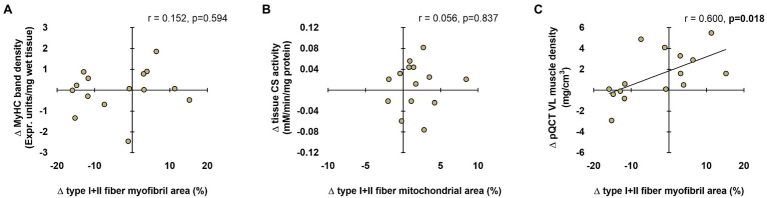
Associations between histology, biochemical, and radiological techniques. These plots demonstrate the relationships between changes in type I+II fiber myofibril area versus change in tissue myosin heavy chain band density (panel A), changes in type I+II mitochondrial area versus change in tissue CS activity (panel B), and changes in type I+II fiber myofibril area versus change in tissue pQCT-derived vastus lateralis (VL) muscle density (panel C). n=15 participants in each panel.

Data in Figure 8 show associations between the various techniques. In short, no significant associations existed between the following variables: (i) change in tissue myosin heavy chain band density from PRE to POST and change in mean (type I+II) fiber myofibril area from PRE to POST (Figure 8A) and (ii) change in tissue CS activity from PRE to POST and change in mean fiber mitochondrial area from PRE to POST (Figure 8B). Interestingly, there was a significant positive association between the change in mean fiber myofibril area from PRE to POST and change in pQCT-derived vastus lateralis muscle density from PRE to POST (Figure 8C).

## Discussion

**Figure 9:**
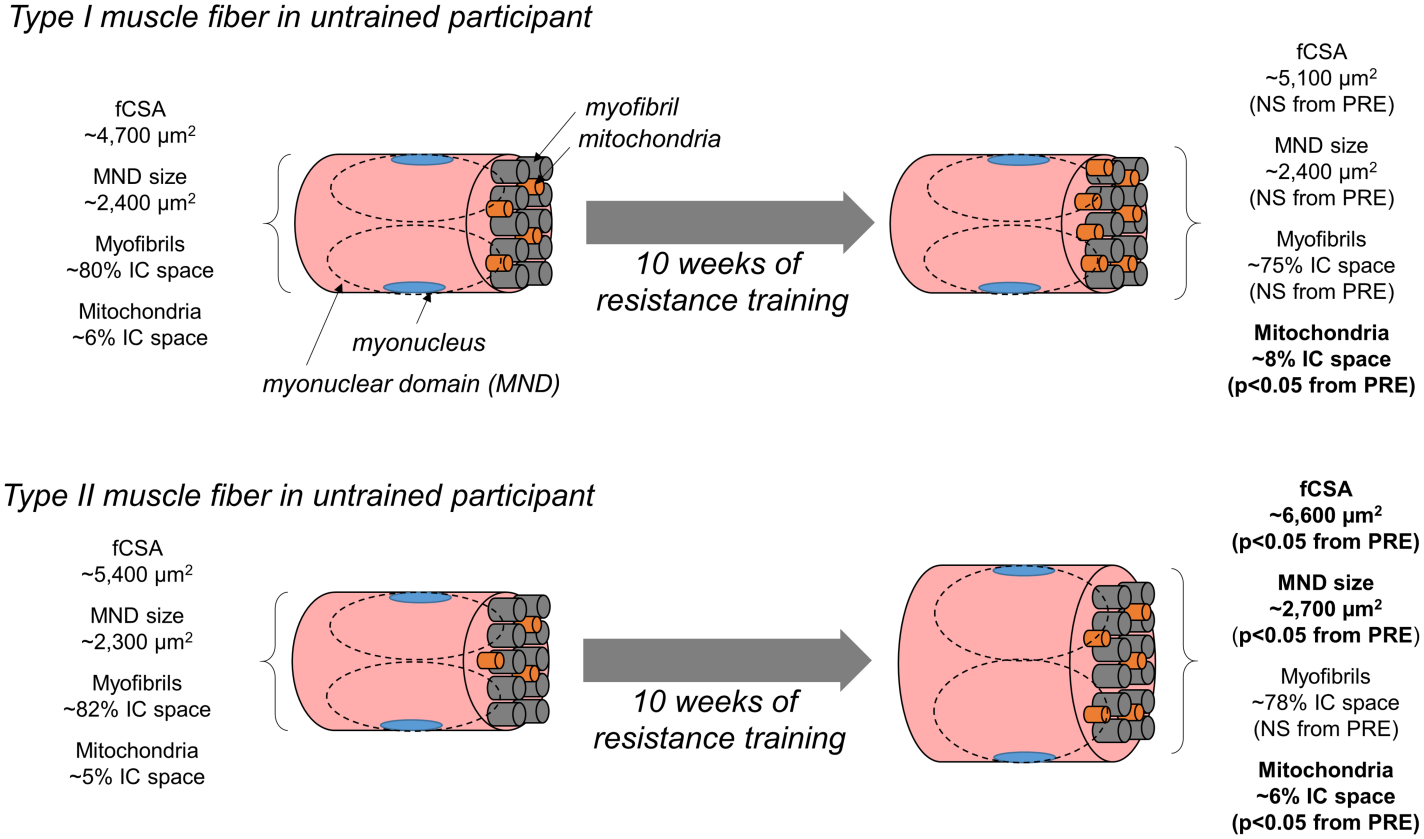
Summary figure of findings. This schematic illustrates our findings. In short, 10weeks of resistance training in untrained men increased type II fCSA (not type I fCSA). Additionally, type II fiber myonuclear domain size increased in type II fibers only. The space occupied by myofibrils did not statistically change in type I or type II fibers. Since increases in type II fCSA occurred, this implies myofibril expansion proportionally occurred with fiber growth. Notwithstanding, we are unsure as to whether this occurred *via* an increase in myofibril number or an increase in the girth of pre-existing myofibrils (not determined herein due to histology limitations). Interestingly, the disproportional increase in the area occupied by mitochondria evidenced through TOMM20 staining suggests the expansion of the mitochondria occurred in type I fibers without fCSA increase, and this expansion occurred more rapidly than changes in fCSA in type II fibers.

Herein, we allocated histology techniques to examine how 10weeks of resistance training affects myofibril and mitochondrial areas in type I and II fibers. In summary, our data suggest myofibril areas within fibers proportionally expanded with increases in fCSA regardless of fiber type. These data suggest conventional hypertrophy, rather than myofibril packing or sarcoplasmic hypertrophy, is the mode through which initial training adaptations occur within a 10-week resistance training period in previously untrained males. Additionally, mitochondrial areas within both fiber types disproportionally increased (i.e., resistance training promoted mitochondrial expansion that outpaced increases in fiber size). A summary of these findings is presented in Figure 9.

We have championed the notion that sarcoplasmic hypertrophy may be a resistance training adaptation (Roberts et al., 2020a). Likewise, we have posited that resistance training may cause a dilution in mitochondrial volume density through the disproportionate increase in muscle fiber size relative to mitochondrial expansion with resistance training (Parry et al., 2020). The current findings contradict these hypotheses and select literature suggesting sarcoplasmic hypertrophy (MacDougall et al., 1982; Haun et al., 2019a) and/or mitochondrial volume density dilution (MacDougall et al., 1979; Tesch et al., 1987; Roberts et al., 2018; Haun et al., 2019a) occurs with weeks to months of resistance training. There are reasons as to why our findings do not agree with these prior reports. First, it should be noted that participants in the two prior studies supporting the sarcoplasmic hypertrophy model were well-trained, and the current participants were not. Moreover, participants in the study by Haun and colleagues engaged in an unconventional and very high-volume training paradigm (Haun et al., 2019a), and a majority of the well-trained participants in the study by MacDougall and colleagues were anabolic steroid users. Thus, if sarcoplasmic hypertrophy is indeed a training adaptation, it remains possible that this phenomenon occurs in well-trained individuals who perform very high-volume training blocks or in individuals that administer anabolic steroids. Regarding the mitochondrial dilution hypothesis, it is difficult to reconcile why this finding was not replicated herein. However, much of these data have been reliant upon using CS activity as a surrogate marker of mitochondrial volume density (Parry et al., 2020). Here, we show that a poor association existed when correlating the changes in mean (type I and II) fiber TOMM20 area and tissue CS activity. Thus, while the CS activity assay may provide a crude assessment of mitochondrial content in tissue homogenates, this method may lack the sensitivity to track changes in mitochondrial volume density with resistance training interventions.

Aside from the aforementioned hypotheses, we have also posited that myofibril density may be directly influenced by myonuclear domain sizes (Roberts et al., 2020a). Specifically, we posited that if fCSA or myonuclear domain sizes became too large with resistance training, this might elicit “transcriptional burden” in resident myonuclei and lead to a disproportionate expansion of non-contractile components. This same logic can also be applied to mitochondria, where a rapid enlargement in myonuclear domain size may hamper the ability of muscle fiber to facilitate mitochondrial expansion. Two moderate negative associations in Figure 5 seemingly agree with our hypothesis and warrant further discussion. First, the data in Figure 5B suggest that the change in type II fiber mitochondrial area was generally not as robust in participants experiencing greater type II fiber hypertrophy. The data in Figure 5D suggest that the change in type I fiber mitochondrial area was generally not as robust in participants experiencing greater increases in type I fiber myonuclear domain size. Both sets of data imply that mitochondrial expansion may be hampered during rapid fiber growth and/or a rapid expansion in the myonuclear domains with resistance training. This makes intuitive sense given that several genes needed for mitochondrial biogenesis and remodeling are expressed from the nuclear genome (Scarpulla et al., 2012). It is also possible that rapidly hypertrophying fibers preferentially synthesize other organelles (e.g., myofibrils and the sarcoplasmic reticulum) prior to mitochondrial expansion during periods of resistance training. Given that these data are limited to correlations, more research is needed to explore the potential relationships between the expansion of the myonuclear domain and mitochondria.

As a secondary analysis, we sought to determine whether the pQCT provided muscle density values that agreed with myofibril density values (i.e., myofibril area values) gleaned from histological analyses. Our motivation for this is that the radiological density of muscle tissue has been previously used to make inferences about the morphological adaptations that occur within muscle fibers. For instance, Claassen et al. (1989) reported 6weeks of resistance training increased Hounsfield units of the mid-thigh by ~4–5% in college-aged men. The authors suggested this was due to either an increase in connective tissue density and/or an increase in contractile protein density. We have also observed that pQCT-derived muscle density significantly increases in older participants following 6–10weeks of resistance training (Lamb et al., 2020) and explained our data in a similar fashion. One manner to view data from the current study is that inferences made from the pQCT are limited given that our histology and electrophoresis data indicated no change in myofibril density occurred with training. However, the significant positive association between the training-induced changes in mean fiber myofibril area and changes in vastus lateralis muscle density do lend credence to pQCT being a viable non-invasive and indirect representation of muscle fiber protein density. Due to the limited n-sizes herein, however, future research is needed to determine the validity of this relationship.

As a secondary analysis, we also sought to determine whether the data from biochemical assays agreed from data obtained from histological analyses. A poor association existed when comparing the training-induced changes in mean myofibril area to changes in relative myosin heavy chain protein content (determined by SDS-PAGE) with training. A poor association also existed when comparing changes in mean (type I and II) mitochondrial area to changes in CS activity levels with training. The latter data are provocative given that CS activity has been widely used as a surrogate marker for mitochondrial volume density (Parry et al., 2020), and this relationship was initially established in 16 males by comparing data yielded from the CS activity assay to data yielded from transmission electron microscopy (TEM; Larsen et al., 2012). This discordance may be due to utilized histological techniques only providing a two-dimensional representation of dozens of muscle fibers, whereas the biochemical techniques assayed characteristics of ~20mg of tissue. Nonetheless, these findings reiterate the notion that data yielded from histological and biochemical techniques may not always agree with one another and need to be viewed independently from one another.

Other interesting findings were evident herein. First, our myofibril data generated through phalloidin-actin staining agree with prior TEM data suggesting myofibrils occupy ~80% of the intracellular space within muscle fibers (MacDougall et al., 1982; Alway et al., 1988). Moreover, these data show that more of the intracellular space is occupied by myofibrils in type II versus I fibers, which also agrees with prior TEM data (Alway et al., 1988). Our TOMM20 IHC data yielded interesting results in that it showed the space occupied by mitochondria was lower in type II versus type I fibers (i.e., a fiber type effect). Moreover, the percent area occupied by mitochondria was (on average) between 5 and 6% at PRE, and these data agree with TEM investigations suggesting the mitochondria occupy less than 10% of muscle fibers in humans (MacDougall et al., 1979; Alway et al., 1988).

### Experimental Considerations

The current data should be viewed with certain limitations in mind. First, the biopsy data are limited to dozens of muscle fibers and to suggest that the results extrapolate to the whole muscle is a bold assumption. Additionally, the matching of type I and type II muscle fibers to serial sections is technically challenging, and this limitation precluded us from capturing more 20x fields of view. Another limitation is that the histological techniques employed herein provide two-dimensional data about muscle fibers. Indeed, this is a larger limitation to most histological investigations in the field. However, Glancy’s group has used three-dimensional scanning electron microscopy techniques to elucidate organelle structures in rodent muscle fibers (Glancy and Balaban, 2021). Notably, these researchers have discovered that muscle mitochondria exist as an interconnected reticulum (Glancy et al., 2015). More recently, this group reported that myofibrils, rather than running parallel to one another, are interconnected to form a lattice/matrix-like structure (Willingham et al., 2020). Hence, employing similar techniques on muscle specimens obtained prior to and following periods of resistance training will further our understanding regarding how various organelles structurally adapt. Finally, our data are limited to younger males, and it is unknown whether these data translate to females or older populations.

## Conclusion

In conclusion, myofibril areas in type I and II fibers proportionally expand with fCSA increases following 10weeks of resistance training (i.e., conventional hypertrophy). Moreover, mitochondrial expansion occurs more rapidly than increases in fCSA, albeit mitochondrial expansion may be dampened in muscle fibers that grow in response to resistance training. The current data also suggest histological and biochemical techniques used to interrogate resistance training adaptations need to be viewed independently from one another given the lack of agreement between the variables assessed herein. Finally, more research is needed to determine whether the pQCT can be used to non-invasively track changes in myofibril density.

## Data Availability Statement

The raw data supporting the conclusions of this article will be made available by the authors, without undue reservation.

## Ethics Statement

The studies involving human participants were reviewed and approved by Auburn University IRB. The patients/participants provided their written informed consent to participate in this study.

## Author Contributions

BR coordinated the training study. BR, JG, and PM performed the molecular assays herein. MR primarily drafted the manuscript, with critical input from BR and JG. All authors were involved with data collection or training participants, provided critical edits, and approved the final version of the submitted manuscript.

## Funding

Funding for participant compensation and assays were provided through laboratory gift donations provided to MR.

## Conflict of Interest

MR receives laboratory funding from various industry sources in the form of fixed-price contracts or laboratory gifts. MR has also been financially compensated by various industry entities for consultation work regarding scientific presentations and/or various scientific writing endeavors in accordance with Auburn University’s Research Compliance and Ethics Guidelines. In relation to the current study, however, none of the authors has financial or other conflicts of interest to report.

The remaining authors declare that the research was conducted in the absence of any commercial or financial relationships that could be construed as a potential conflict of interest.

## Publisher’s Note

All claims expressed in this article are solely those of the authors and do not necessarily represent those of their affiliated organizations, or those of the publisher, the editors and the reviewers. Any product that may be evaluated in this article, or claim that may be made by its manufacturer, is not guaranteed or endorsed by the publisher.
